# A review of the United States global surgery program landscape by website analysis

**DOI:** 10.1186/s12893-025-02979-6

**Published:** 2025-09-01

**Authors:** Lauren E. Cox, Cassandra M. D’Amico, Shriya Bhoothapuri, Joseph A.Q. Karam, Rachel W. Davis, Mike M. Mallah

**Affiliations:** 1 College of Medicine, Medical University of South Carolina, Charleston, SC USA; 2 Department of Surgery, Medical University of South Carolina, Charleston, SC USA; 3 Global Surgery Program, Medical University of South Carolina, Charleston, SC USA; 4 Center for Global Surgery, Baylor College of Medicine, Houston, TX USA

**Keywords:** Global surgery, Program development, Education, Research, Service, Bidirectionality, Partnerships

## Abstract

**Background:**

Over the past decade, since the 2015 Lancet Commission on Global Surgery (LCoGS) highlighted the global burden of disease attributable to a lack of safe surgical care, medical degree-granting institutions across the United States (US) have worked to increase engagement in global surgery. The research team aimed to analyze the current landscape and provide an overview of all US-based global surgery programs. It was predicted that most medical institutions in the US would not have established programs. For those with global surgery programs, their mission statements and demonstrated output were classified according to a list of five domains, including bidirectionality, education, partnerships, research, and service. These domains were generated from the priorities outlined by the LCoGS 2030 objectives as there is no universally accepted gold standard for quality evaluation in global surgery education. The team hypothesized that mission statements for existing programs would meet a majority, but not all, of the five domains, and that programs would demonstrate less output than their projected goals.

**Methods:**

The team conducted a qualitative analysis of all global surgery programmatic offerings across the US. A list of terms was established to analyze the websites published for each US allopathic (MD) and osteopathic (DO) program. An Excel matrix was produced that outlined all desired information. The domains were used to organize and classify the collected data.

**Results:**

Out of 194 US MD- and DO- granting institutions, 39 had global surgery programs. Twenty-five programs had missions that addressed three to four of the domains and 12 programs projected pursuit of all five domains. Of the 12 programs that projected this mission to meet all five objectives, six demonstrated tangible output in all five areas. Bidirectionality was the most common domain not addressed by programs in either their mission statement or output.

**Conclusions:**

Global surgery is a nascent field, and as predicted, the majority of medical institutions do not have a global surgery program. Furthermore, institutions with programs and well-defined missions did meet a majority of the five domains. Contrary to the team’s prediction, most existing programs demonstrated equal or greater output than their expressed goals.

## Introduction

The increasing prominence of global surgery in international health dialogue, infrastructure development, and educational programming highlights a growing recognition of the crucial roles surgical capacity and collaboration play in addressing broader global health challenges. While communicable disease burden appears to be declining, noncommunicable diseases, of which trauma and emergency surgery are a component, account for nearly one-third of the entire global burden of disease [1]. Five billion people across the world lack access to timely surgical and anesthesia care, and nine out of ten of those are from low- and middle- income countries (LMICs) according to the Lancet Commission [1, 2]. The World Bank’s Disease Control Priorities 3 (DCP-3), specifically Volume 1 of *Essential Surgery*, also identify 44 surgical procedures considered essential for public health, based on addressing substantial global need, cost-effectiveness, and ease of implementation [1, 3]. Despite this push for prioritization of surgery and anesthesia, systems are developing too slowly to meet population needs by 2030 [2]. 

With the recognition of the barriers that underdeveloped systems and limited access to surgery create for ill and injured patients, the World Health Organization (WHO), World Bank, and Lancet Commission on Global Surgery have all called for safe surgery prioritization worldwide [1]. In 2015, the LCoGS crafted a list of objectives to achieve by 2030 including 80% coverage of essential surgery and anesthesia across 110 country collaborators [1, 4]. In 2015, a WHO resolution to strengthen emergency and essential surgical care and anesthesia as a component of universal health coverage was unanimously approved at the 68th World Health Assembly.

As a result of this interest, global surgery began to emerge as an academic discipline, with many residencies offering international clinical residency and fellowship rotations. These rotations aimed to educate and provide exposure to surgery in resource-variable contexts to better prepare learners to meaningfully improve access gaps. Furthermore, many US academic institutions and specialty groups aimed to develop global surgery programs, centers, institutes, or student organizations to further the global agenda. One organization, the Global Surgery Student Alliance (GSSA), serves to connect medical students with the global mission set forth by the Lancet Commission. Beyond serving their own members, many of these programs have contributed to LMIC training, advocacy, surgical research capacity, innovation, and health system strengthening through provision of resources, advocacy, research, awareness, and support [1, 2]. Virtual educational tools have also been established in an effort to implement greater training opportunities in low-resource settings [5]. 

Despite the efforts made since 2015, there are still many areas for progress and improvement. Even with the rise in interest and efforts, a lack of generalized and shared understanding remains in the field of global surgery. Therefore, the team aimed to produce a comprehensive review of all global surgery programs within the US. The purpose of this study was three-fold. First, the team aimed to identify the global surgery program landscape across US academic medical institutions. Second, this study aspired to classify the mission of each program using five domains generated by the research team based on priorities outlined by the Lancet Commission objectives to meet by 2030: bidirectionality, education, partnerships, research, and service. Third, the team planned to categorize the output of each program according to these five domains and compare them with their respective mission statements. The team hypothesized that most academic medical institutions across the US would not have an established global surgery program. Furthermore, it was predicted that for the institutions that did have a program, most projected missions would meet a majority — three to four — of the five domains, and the demonstrated output of each program would be less than the projected output.

## Methods

**Fig. 1:**
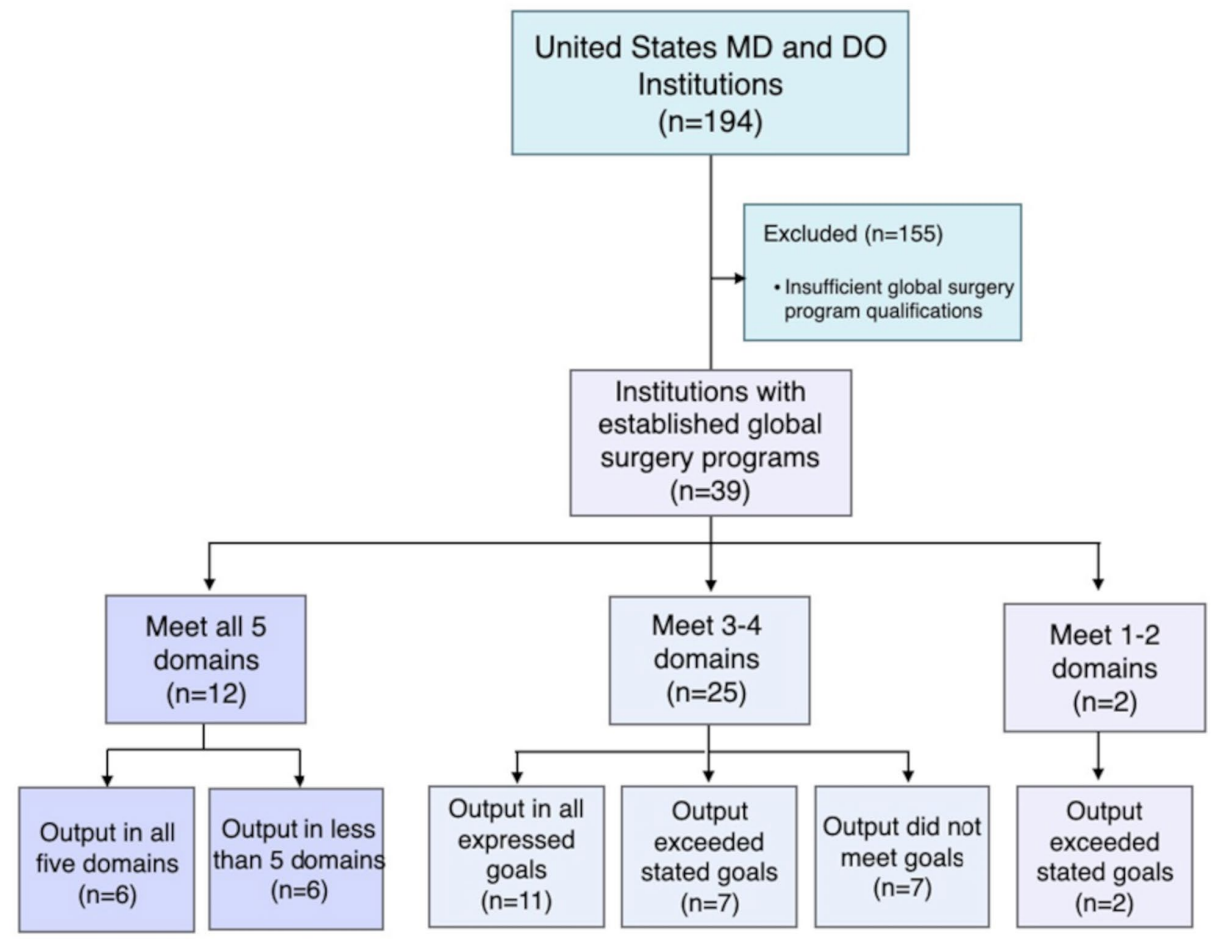
Breakdown of inclusion criteria and programs classified

The primary research team is based at a large academic medical center in Charleston, South Carolina. To reduce institutional bias, a second academic medical center was engaged for review. The team conducted a qualitative program analysis of all MD- and DO- granting institutions across the US with a global surgery program. A comprehensive list of all MD and DO programs was obtained from the Association of American Medical Colleges and the American Osteopathic Association [6, 7]. This list identified 194 MD and DO schools in the US, which is depicted in Fig. 1. A list of key terms was generated pertaining to global surgery. Each institutional website was searched for the outlined terminology. To search these webpages, the team used the search function on the institution’s main page to scan for the terms included in Table 1 and to identify the global surgery engagement level. Only schools with an established global surgery program were included in this study. A global surgery program was defined as a center, institute, or program with a disclosed mission and recognition by the respective university on a webpage. Programs with a single global surgical element, such as an elective, service trip, or GSSA chapter, were not considered a formal global surgery program and therefore were not considered in this study. The 39 schools that met these criteria were included in the second phase.(Fig. 1) The team then used the search function to glean for specific terminology on program webpages as described in Table 1. Each term had associated keyword criteria that assisted in its categorization. For example, if a website indicated the keyword “instruct,” this led to its categorization under the education component.

**Table 1:** Term criteria classified in mission statements as “meeting” term component

Term	Associated Keyword Criteria
Global Surgery	“global surgery (‘program’ OR ‘center’ OR ‘institute’)”
Mission Statement	“mission statement” “objectives” “goals” “aims”
Education	“educate” “instruct” “teach” “guide" “train”
Research	“research”
Partnerships	“partner” “partnerships” “relationships”
Service	“service” “advocacy” “capacity building” “volunteerism”
Bidirectionality	“bidirectional” “visiting partners” “exchange”

The team outlined criteria in the matrix for systematically collecting data and analyzing each program, including questions pertaining to five domains that address many priorities outlined by the Lancet Commission’s 2030 goals: bidirectionality, education, partnerships, research, and service [4]. Bidirectionality is defined as engagement between partners, specifically with ideas, resources, and personnel exchanges occurring in both directions. Education is recognized as instruction, training, or knowledge sharing through seminars, courses, or curricula. Partnership is the establishment of a relationship between two institutions. Research is the investigation of unknown topics within a field, and the compilation of findings within some final product. Service is defined as the provision of assistance in some capacity to a party that is in need, whether through direct actions, indirect involvement such as fundraising and program coordination, or advocacy to raise awareness.

Each program was first classified by whether its mission outlined goals to achieve each of these five key areas. This was executed by using the terminology outlined in Table 1 to find relevant webpages, which were then analyzed to see if the content answered the questions pertaining to each domain included in the matrix.

**Fig. 2:**
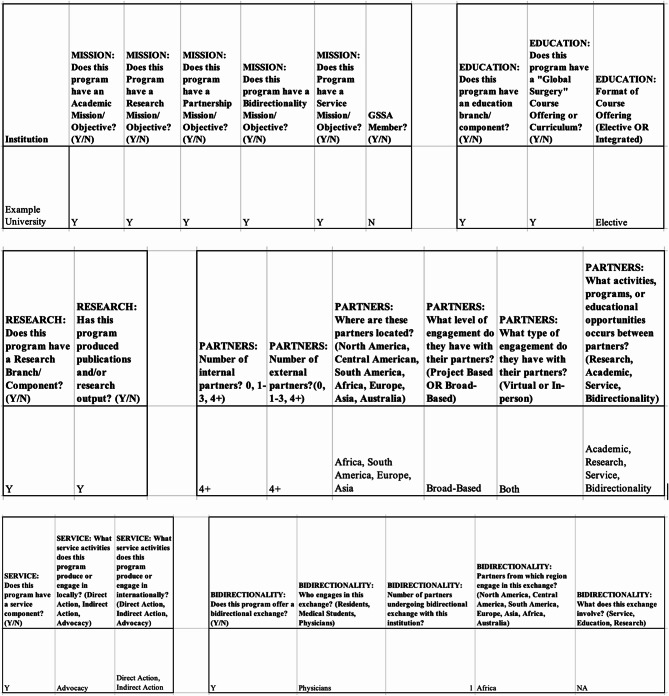
Example of matrix data collection

Once each individual programmatic mission was analyzed, the team proceeded to investigate whether the program demonstrated tangible output in these domains. Table 2 outlines the terminology perceived as “output” under each domain. An example of the data that were collected for each institution and under each domain is showcased in Fig. 2.

**Table 2:** Evaluation criteria of each domain

Domain	Fields Classified
Education	course offering or curriculum
Research	publications AND/OR research output
Partnerships	number of internal partners; number of external partners; partner locations; project based or broad-based partner engagement; virtual or in-person partner engagement; activities, programs or educational opportunities between partners [service, education, research]
Service	local service engagement [direct action; indirect action; advocacy]; international service engagement [direct action; indirect action; advocacy]
Bidirectionality	bidirectional exchange; who engages in exchange; number of partners undergoing exchange; location of exchange; what does this exchange involve [service, education, research]

From these data, programs were further investigated to determine how many both projected a mission to meet all five domains and demonstrated tangible action items in each of these domains.

## Results

### Broad programmatic overview

Thirty-nine of 194 US MD- and DO- granting institutions had established Global Surgery Programs. Thirty-eight of the 39 programs were from MD-granting institutions, as shown in Table 3. Twenty-eight of them were Department of Surgery programs, and the remainder were programs within Centers for Global Health, student organizations, or stand-alone institutes, centers, or programs. Nine programs were located in the Central US region, six in the Northeast, ten in the Mid-Atlantic, eight in the West, and six in the South. Twenty of the institutions were private, and the remainder public. Sixteen of the programs had interdisciplinary involvement, meaning they engaged fields beyond medicine, including physician assistants, nursing, dentistry, pharmacy, physical therapy, master’s programs, and doctoral (PhD) programs. Twenty-seven programs revealed student engagement in addition to graduate-level engagement including residents, fellows, and attendings. (Table 3) Twenty-seven of the programs classified have GSSA membership. Seven of the 12 programs classified that do not have student involvement also do not have GSSA membership.

**Table 3:** Overview of every global surgery program in the US

Characteristic	*N* = 39	%
**Degree-Seeking Institution**		
MD	38	97.4%
DO	1	2.6%
**Program Affiliation**		
Department of Surgery	28	71.8%
Global Health Center or stand-alone programs including student organizations	11	28.2%
**Regions**		
Central	9	23.1%
Northeast	6	15.4%
Mid-Atlantic	10	25.6%
West	8	20.5%
South	6	15.4%
**Institutional Funding**		
Private	20	51.3%
Public	19	48.7%
**Interdisciplinary Involvement**		
Yes	16	41.0%
No	23	59.0%
**Involvement**		
Students	27	69.2%
Residents	34	87.2%
Fellows	22	56.4%
Physicians/Faculty	36	92.3%

### Programmatic mission evaluation

Programmatic missions were divided into three evaluation categories based on number of the following domains addressed: bidirectionality, education, partnership, research, and service. Programmatic missions were classified as incorporating all five domains, three to four domains, or one to two domains. Twelve programs met all five domains, two programs met one to two domains, and 25 programs met three to four of the domains. The most common domain that was not addressed by the mission of programs was bidirectionality, with 20 programs lacking a bidirectionality mission.

### Programmatic output evaluation

After evaluating missions and projected goals, the team analyzed the stated output of each program. Programs were classified as having met all five domains, three to four domains, or one to two domains. Twenty-five of the programs executed three to four of the domains, 13 programs executed all five domains, and only one program executed one to two of the domains. The most common domain that was missing from program action items was bidirectional exchange, with only 25 of the programs having demonstrated this.

**Fig. 3:**
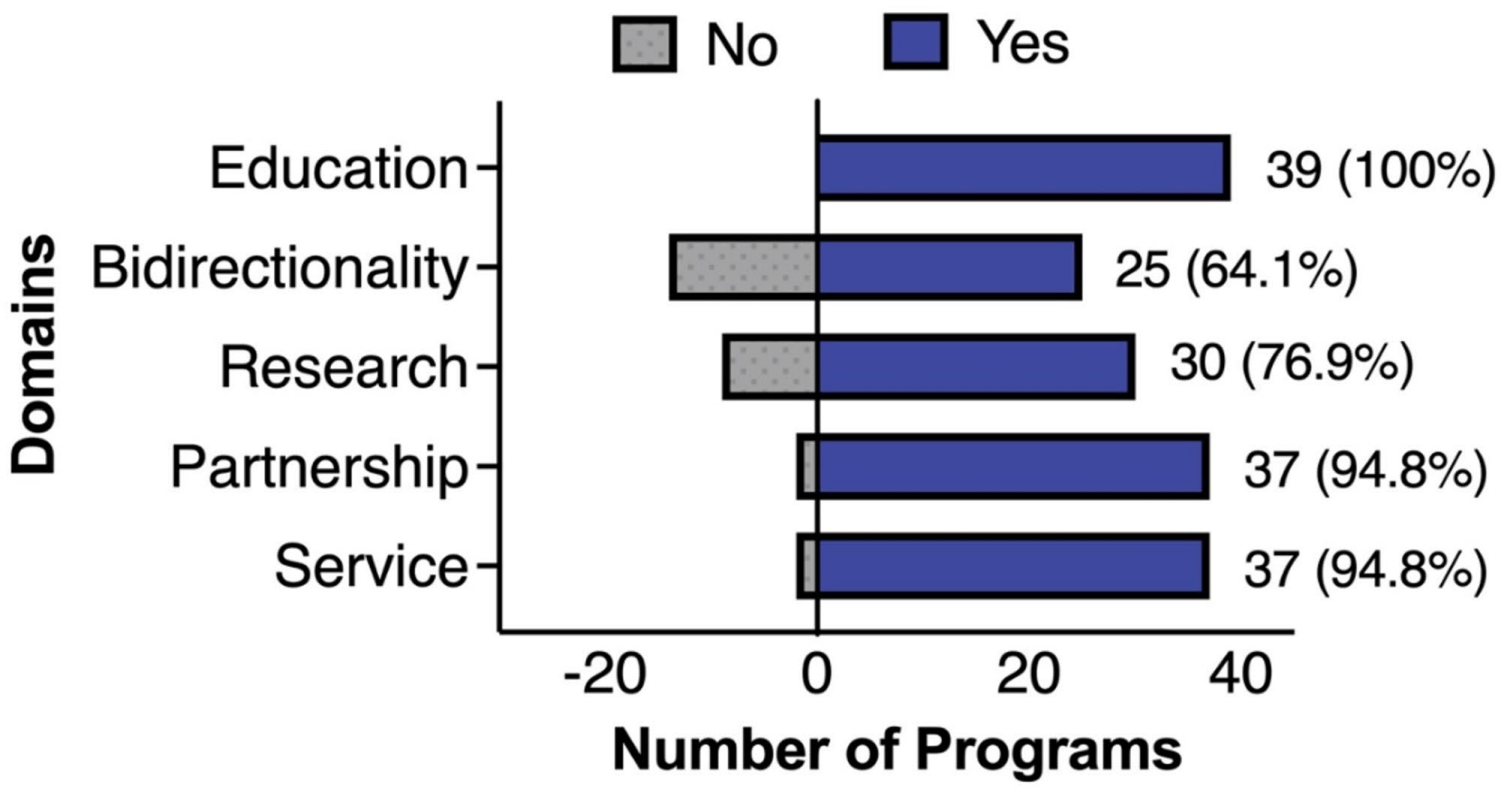
Overview of core domains met by output of each global surgery program. Denoted by either “yes” or “no”

In analyzing programmatic output, the team found that all programs expressed an educational element, which is reflected in Fig. 3. Programs listed as having educational components mainly satisfied this element by directing their teachings to the local institution, as opposed to the partners in which they serve. Twenty-four of the 39 programs offered courses, all offered electives, and nine were longitudinally incorporated into the curriculum. Thirty of the 39 programs had a research component, 18 having produced publications, found through institutional websites and PubMed. (Fig. 3) Programs were classified as having “internal” or “external” partnerships depending on whether they were engaged with partners found within or outside of the respective institution. Seven of the 39 programs identified one internal partner, 13 had greater than four internal partners, and two had no internal partners. Twenty-seven of the programs had greater than four external partners and ten had one to three external partners. Eleven had partners in North America, 15 in Central America, 17 in South America, 31 in Africa, 22 in Asia, and ten in Europe. All programs with partnerships except one had broad-based relationships with their partners, and 27 programs had both virtual and in-person interactions with their partners. Twelve of the 39 programs interacted with their partners in education, research, service, and bidirectionality, whereas the remainder of programs with partnerships participated in fewer than four of those fields.

Thirty-seven programs had a service component. Service is described as direct action (action taken to create a concrete effect on an issue), indirect action (fundraising, donations, or organizing a program), or advocacy (speaking on behalf of an issue or solution) [8]. Nine of the programs had local service projects; five of these projects engaged in direct action, six via indirect action, and eight through advocacy. Thirty-seven of the programs had foreign service engagement, 35 of which involved direct action, 30 through indirect action, and 21 through advocacy. Twenty-five programs had bidirectional exchange.(Fig. 3) Ten of these exchange programs involved faculty, nine involved residents, and six involved medical students. Six of the programs had bidirectional exchange with partners in Africa, four in Asia, two in Central America, four in Europe, two in South America, and one in North America. Through this exchange, 12 of the programs engaged in education, four in research, and three in service.

### Comparison of program mission to demonstrated output

In comparing programmatic mission statements to demonstrated output as described on webpages, 50% of the 12 programs with mission statements incorporating all five domains also met demonstrated output of all five. The other half did not show demonstrable actions in the fields of service, research, or bidirectionality. A domain was categorized as “action met” if the program demonstrated concrete effort in this domain. For instance, the education component was designated as “action met” if they offered a global surgery course or curriculum. The service domain, as mentioned previously, included direct, indirect, and advocacy and therefore, any actionable steps toward either type of service was categorized as “action met”. As depicted in Table 4, there were 20 programs that expressed a mission to pursue all domains except bidirectionality, 11 of which met those expressed goals through tangible actions, seven exceeded through completing action across all five domains, and two did not demonstrate action to meet their goals. There were two programs that projected missions to meet only one to two domains and demonstrated output that exceeded these goals.(Table 4) In total, only eight programs projected missions to achieve multiple goals that they did not meet or exceed.

**Table 4:** Comparison of programs mission statement and action of domains

Global surgery overview		
**Characteristic**	**Listed in Mission**	**Action Met**
**Domains**		
5	12	13
3 to 4	25	25
1 to 2	2	2
**Bidirectionality**		
Yes	19	25
No	20	14

## Discussion

Global surgery programs at medical degree-granting institutions have been designed to better equip physicians to address the global lack of access to surgical care, improve patients’ quality of life, and promote health and equity. The findings of this study may encourage programs to consider their programmatic missions and academic outputs within the field of global surgery. Eight of the identified programs did not express outputs on their websites consistent with their foundational mission and goals. This may be a reflection of incomplete web content, barriers to enacting their goals, or generalized lack of resources. Some academic global surgery programs may benefit from achieving these goals from interinstitutional and organizational support networks.

This review supports and aligns with several existing studies in the current literature [8–10]. The observed gap in bidirectionality reinforces the existence of an opportunity imbalance that persists between HICs and LMICs. Bidirectional frameworks have been identified by previous studies as being a key element for training and accrediting international surgeons, in addition to emphasizing culturally specific competencies, standardizing core surgical skills, and decentralizing global systems [10]. However, national and state legislative barriers including limitations in licensing and visas remain a significant challenge for many programs. Additionally, this study defined bidirectionality as exchange in two directions. This narrow interpretation should be recognized as limiting. Previous studies have demonstrated a lack of bidirectional engagement in multiple areas. For example, one study found that increased commitment to the improvement of global surgical care has not yet translated to equal exchange of surgical information between HICs and LMICs [5]. This observed gap in bidirectional involvement informed the focus of our study and underscores the need to enhance bidirectional research, innovation, and education moving forward.

The rise of innovative technologies and systems that foster engagement also increases the avenues through which global surgery programs can achieve their goals. Given findings that several domains have not yet been comprehensively enacted across global surgery programs, it may be beneficial to adapt existing technologies to align with the resources and specific needs of each region. These technologies should be strategically implemented to maximize their impact, ensuring that students, residents, and physicians across resource levels benefit from tailored, effective education. For example, bidirectional educational training opportunities can be expanded through virtual curricular models, simulators, and immersive technologies [5]. 

This study also identified that nearly all of the programs classified have at least one external partnership, and that all of these partnerships except one could be classified as broad-based, which the team defined as a relationship extending beyond the scope of a single project. Several studies have highlighted the importance of program and relationship sustainability, and have emphasized the use of formalized multilateral agreements to do so [10]. 

It is important to recognize the limitations of this study. The data were collected from publicly accessible information on institutional websites, including program details and updates. If actions or achievements were not published on the websites before January 2025, they were not included in this study. This is a significant limitation, as the majority of the study’s data analysis relied on information presented on these institutional webpages, which may have influenced the results and interpretation of global surgery programs. Additionally, the team recognizes potential confirmation bias may have influenced the curation of the five domains. The risk of coding bias is also acknowledged in the data analysis process. In collecting information from institution websites, this study aimed to remain objective through development of standardized lists of terminology. However, this system does not eliminate all risk of researcher bias. The team attempted to further minimize researcher bias through partnership with a second large academic medical center. Given the limitations posed by data collection from websites, the team will pursue a follow-up study to collect information directly from each institution through a survey and perform a thorough program evaluation. The goal of the follow-up study will be to bridge the gap in information and compensate for missing data and inaccuracies that may be present on institutional websites.

Significant gaps remain in the literature, especially pertaining to the economics of global surgery, capacity building, and encouraging community resilience through local partnerships and contributions. These gaps inhibit complete understanding of the extent of the challenges that hinder the establishment of effective global surgery programs worldwide. Future research should continue to investigate these understudied areas. The team intends to expand upon the findings of this study to classify whether there are certain programs with overlapping partnerships and projects.

Our study provides an analysis of global surgery programs within the United States, offering insights into the development and progress made by these programs. The US-centric approach is intended to provide a clear, methodologically rigorous foundation that can be expanded to include global surgery centers in other HICs as well as LMICs. Understanding the evolution of global surgery in LMICs is particularly crucial to assessing the specialty’s impact and offers an opportunity to foster partnerships with LMIC institutions. Global surgery programs connect US-based institutions with the international community through the goal of reducing the burden of non-communicable diseases and improving surgical capacity worldwide. By elucidating the current state of academic global surgery education, it is the team’s hope that this study can encourage programs to periodically classify their missions and output, ensuring efforts continue to drive meaningful progress.

## Conclusions

The team accurately predicted that most academic medical institutions across the US (79.89%) do not have an established global surgery program. Additionally, most programs (64.10%) projected missions that meet a majority — three to four — of the five domains explored. However, in contrast to expectations, it was found that most global surgery programs met or exceeded their projected output. Bidirectionality is the least represented domain across programs, which can be better recognized and addressed in the future.

These results suggest that many programs aspire to meet the global surgical and anesthesia needs highlighted by the Lancet Commission. However, programs may encounter barriers to meeting these goals. Furthermore, these actions could benefit from greater visibility through institutional websites and publications to increase public awareness and aid in collaboration.

## Data Availability

The datasets used/and or analyzed during the current study are not publicly available due to placing the authors in a potentially compromising position with the institutions on which they report, but are available from the corresponding author on reasonable request.

## Abbreviations

US: United states
MD: Allopathic
DO: Osteopathic
LMIC: Low and middle income countries
DCP-3: Disease control priorities 3
WHO: World health organization
LCoGS: Lancet commission on global surgery
GSSA: Global surgery student alliance
